# Mergers and bank branches: two decades of evidence from the USA

**DOI:** 10.1007/s00181-022-02307-4

**Published:** 2022-11-17

**Authors:** Joan Calzada, Xavier Fageda, Fernando Martínez-Santos

**Affiliations:** 1 Department of Economics and BEAT, University of Barcelona, Av. Diagonal 696, 08034 Barcelona, Spain; 2 Department of Applied Economics and GIM-IREA, University of Barcelona, Av. Diagonal 690, 08034 Barcelona, Spain; 3 Department of Economics, Universidad Carlos III de Madrid (UC3M), Calle Madrid, 126, 28903 Madrid, Spain

**Keywords:** Bank branches, Mergers, Broadband, Financial exclusion, Great Recession, L16, L22, G21, G34, G38

## Abstract

In recent decades, the US bank market has been exposed to several waves of mergers, resulting in concerns about branch presence and consumer access to financial services. This paper examines the effects of bank mergers on branch density in the period 2000–2020. To do so, we use panel regressions and matching techniques at the census tract level to study the impact of inter- and intrastate mergers before and after the Great Recession of 2007. To generate plausible exogenous variation for mergers, our analysis focuses on transactions involving large entities, and we consider the within-tract variation in exposure to mergers. A comparison of exposed and unexposed tracts shows that in the period under study each merger  reduced branch density by around 3%. Moreover, interstate mergers reduced branch density at the tract level across the whole period but had an expansionary effect on the number of branches at the county level before the crisis. Intrastate mergers, in contrast, had a consolidation effect across the whole period, an impact that was more intense in rural tracts and in tracts where merging entities operated overlapping branch networks. Finally, we show that the reduction of bank branches was stronger in tracts with a relatively higher penetration of broadband Internet services, but we find no evidence that the adoption of FinTech services intensified branch closures.

## Introduction

In recent decades, the US banking market has undergone a notable process of consolidation that has reduced the number of banking entities and transformed their branch networks. Market liberalization, introduced by the *Riegle-Neal Act* of 1994, favored the expansion of banking entities into other states and contributed to the increase in the number of bank branches. This situation was reversed after the 2007–2008 financial crisis and the Great Recession. Between 2009 and 2020, fifteen per cent of branches were lost nationally, a trend that is set to continue in the years ahead due to the increasing digitalization of the banking sector, the emergence of FinTech services and changes in consumer habits in the wake of the coronavirus pandemic. Urban areas have suffered high levels of closures and ‘banking deserts’ are today familiar features of rural areas. All in all, this process has raised concerns that a growing number of households and businesses might be losing adequate access to financial services. Branch closures increase consumers’ financial services costs, such as cashing checks, obtaining loans or simply opening deposit accounts. They are, moreover, forced to travel longer distances to contract such services, while losing the personal advisory services they received from their traditional bank tellers due to staff reorganizations. An additional concern is that this process of ‘financial exclusion’ may be more intense in areas with higher proportions of low-income families and minorities, groups that frequently have limited mobility and lower rates of Internet access (Sinclair 2001; Carbo et al. 2007; Degryse and Ongena 2005; Ergungor 2010; Nguyen 2014).^1^

Bank branches play a fundamental role in the functioning of the banking sector. Despite the evolution in technology and the widespread adoption of online banking, brick-and-mortar offices remain a primary mechanism of contact between customers and financial agents (FDIC 2018). Bank branches allow customers to undertake various business dealings that have yet to be replaced by online banking, including cash transactions, obtaining financial advice and contracting services such as credit facilities and insurance products. In this context, the closure of branches in certain locations hinders the access of consumers to financial services. Banks also lose out as a result of these closures as their employees generate less soft information, but this effect can be offset by the reduction in the cost of premises and the introduction of new practices for screening customers.

The commercial strategy of banking entities has changed notably in recent decades. The 1994 *Riegle-Neal Interstate Banking and Branching Efficiency Act* promoted unrestricted nationwide banking and branching activities and favored the use of mergers to gain access to new markets.^2^ In the nineties, non-bank competitors (such as in-store supermarket bank branches) emerged as an alternative means for providing financial services. As a result, large banks saw the *Riegle-Neal Act* as an opportunity to expand into other states to gain scale, and to further diversify and consolidate their position (Elfakhani et al. 2003; Allen et al. 2014). At the same time, the adoption of digital technologies—which allowed banks to reduce their operating costs and improve their information systems—emerged as a key factor in the transformation of the sector. Indeed, those banks that could not afford to invest in new technologies became targets for acquisition.

The path of evolution taken by the sector shifted notably during the Great Recession. After 2007, a series of regulations were introduced aimed at providing consumers with greater protection and preventing future crises. At the same time, the reduction in the banks’ profitability due to the slump in demand and falling interest margins led to new mergers in the sector. These mergers ushered in numerous branch closures that helped banks reduce their costs and intensified the expansion of online banking.^3^

This article examines the transformation of the brick-and-mortar branch structure in the US banking system in the period 2000–2020. Specifically, we seek to analyze the effects of bank mergers on branch density in different geographical areas of the country, before and after the Great Recession of 2007. The period chosen for our analysis is useful to examine how banks have used mergers to adjust to different economic and regulatory environments. For the pre-crisis period, we consider the years from 2000 to 2007, characterized by significant economic expansion; for the post-crisis period, we consider the years of economic crisis and subsequent recovery up to 2020.

Our analysis draws on a rich dataset for 72,738 census tracts located in the 50 states of the USA and the District of Columbia. Information about bank branches at the tract level has been obtained from the Federal Deposit Insurance Corporation (FDIC). Tract-level demographic and socioeconomic variables are taken from the US Census Bureau.

One obvious difficulty in estimating the effects of mergers on branch closures is that a merger might be influenced by the prevailing economic conditions of local markets. To address this possibility, we estimate a fixed-effects model that analyzes the impact of mergers on branch closures and openings at the census tract level. Following Nguyen (2019), our estimation strategy exploits within-tract differences in exposure to mergers and we consider county-by-year fixed effects to control for specific time trends at the county level. We consider that mergers are plausibly exogenous to local economic conditions and compare economically similar census tracts exposed and unexposed to mergers. To further ensure that banks’ merger decisions are exogenous to the situation of local markets, we restrict our analysis to mergers between large banks (Nguyen 2019; Jagtiani and Maingi 2019). Specifically, we consider 106 mergers involving entities with more than $1 billion in pre-merger assets and where the acquired bank operated more than 80 branches. Moreover, we focus on mergers between banks in different states (interstate mergers) and between banks in the same state but with their headquarters in different counties (intrastate/inter-county mergers). Finally, we complement this identification strategy with the application of a matching process to select a more comparable group of tracts exposed and unexposed, respectively, to mergers. Specifically, we use a propensity score matching algorithm to individually assign to those tracts affected by mergers other comparable tracts unaffected by such transactions. The variables included in the matching analysis are the socioeconomic characteristics of the tracts.

Our results indicate that during the period 2000–2020 each merger between large banks reduced branch density by 3% in those tracts in which the acquired bank had a presence and that this effect increased up to 6.5% in those tracts in which the acquirer and the targeted banks had overlapping branch networks. Interstate mergers reduced branch density by 2.7%, while intrastate mergers reduced the density by 4.5%.

In the years before the Great Recession and in the first decade of the new century, branch density increased notably across the USA. While in 2000 there were 85,492 bank branches, by 2009 this number had climbed to 99,550. In this period, interstate mergers had an expansionary effect and branch density increased in the affected counties; but, in tracts directly exposed to mergers, the increase in branch density was not so great. This outcome reflects the consolidation behavior adopted by the acquiring entities, especially in tracts with overlapping branch networks. After the crisis, banks initiated a major *de-branching* policy and by 2020 the number of branches had fallen to 85,050. Interstate mergers contributed to this adjustment at the tract and county levels and in tracts with overlapping branch networks, the density fell 5% in comparison with that in unexposed tracts.

Intrastate mergers, meanwhile, had a negative effect on branch density both at the tract and county levels across the whole period under study. More specifically, after the financial crisis, intrastate mergers had a strong impact on rural tracts and tracts in which the merging banks had overlapping branch networks. Thus, large bank mergers reduced branch density by 14% in rural tracts and by 12.6% in tracts with network overlaps.

Our paper also shows that, over the last decade, the consolidation effect of mergers has been stronger in tracts with a relatively higher penetration of broadband Internet. This outcome suggests that online banking serves as a substitute for brick-and-mortar branches and that the deployment of high-capacity telecommunication networks can accelerate the closure of bank branches.^4^ However, our analysis also reveals that the adoption of FinTech services, proxied here by the percentage of mortgage lending covered by FinTech at the county level, has not intensified de-branching. A possible explanation for this outcome is that online mortgage lending remained relatively underdeveloped in the period of years we have examined.^5^

Several papers have analyzed the branching strategy of banking entities in the USA. While some authors have focused on the effects of branch competition on prices (Sapienza 2002), others have considered its implications for service quality (Focarelli and Panetta 2003; Panetta et al. 2009), firm–bank relationships (Karceski et al. 2005; Panetta et al. 2009), consumer bankruptcies (Allen et al. 2016), and the efficiency and the stability of bank entities (Calomiris 2000; Aubuchon and Wheelock 2010; Aguirregabiria et al. 2016). There is also a line of literature examining the determinants of branch expansion and new charter formation. Amel and Liang (1997) analyze over 2000 US banking markets in the period 1977–1988, showing that bank entry is determined by population growth and incumbent profits. Adams and Amel (2007) study bank entry from 1994 to 2008 and find that entry is positively associated with local demand and past entry and negatively associated with incumbent branch expansion and small bank presence. Cohen and Mazzeo (2010), in an examination of the consequences of the sectorial reforms introduced in the nineties in the US rural bank market, show that while competition from traditional single-market banks and saving banks is associated with smaller branching networks, all types of institutions tend to extend their branch networks when competing with multimarket banks.

The main contribution of our study to this literature is to show the heterogeneous impact of mergers on branch deployment across different periods and different types of socioeconomic area. Specifically, we examine the impact of large mergers during pre- and post-crisis periods at the tract level and analyze the differential effects for urban and rural areas. Additionally, we consider the differential effect of interstate mergers (i.e., where the merging entities had their headquarters in different states) and intrastate/inter-county mergers (i.e., where the merging entities had their headquarters in the same state). We consider that inter- and intrastate mergers may have had different impacts because they had different motivations. While one of the objectives of the former was to expand into other markets (especially prior to the Great Recession), the objectives of the later were to generate efficiency gains and cost reductions.

Our paper is also related to the literature analyzing the effects of branch deployment on financial exclusion. Branches can alleviate information frictions by collecting soft information about consumers and their neighborhoods. In this sense, Celerier and Matray (2020) exploit the staggered interstate branching deregulation in the USA after the *Riegle-Neal Act* as an exogenous shock on bank competition and show how the competition resulting from these regulations reduced the share of unbanked households, benefiting those with lower income and living in rural areas. Other papers have shown that the distance between lenders and borrowers determines the availability of and the terms of credit, especially in low-income neighborhoods (Degryse and Ongena 2005; Agarwal and Houswald 2010; Ergungor 2010; Brown 2016; Allen et al. 2012; Beck et al. 2019; Martin-Oliver 2019). There is also a relevant line of literature highlighting the relationship between mergers and financial exclusion. Allen et al. (2014) analyze how a merger between Toronto Dominion and Canada Trust in 2000 affected consumer bankruptcy. Beck and Martínez Peria (2010) examine the effect of foreign bank acquisitions on banking outreach in Mexico in the period 1997–2005.^6^ Jagtiani and Maingi (2019) investigate the impact of mergers affecting community banks on local small business lending in the period 2002–2014.^7^ Nguyen (2019) analyzes the level of lending to small firms in neighborhoods exposed to the merges of large banks in the period 1999–2012, showing that branch closures caused by the mergers lead to prolonged declines in local small business lending.

The rest of the paper continues as follows: Section 2 describes the main episodes in the US banking market before and after the Great Recession. Section 3 describes the dataset, outlines the main market trends, and explains our empirical strategy. Section 4 presents the results of our analysis. Finally, Sect. 5 concludes.

## The US banking market before and after the Great Recession

Banking deregulation has been a major determinant of the deployment of bank branches since the end of the 1990s. In the early century, the *McFadden Act* of 1927 implicitly prohibited commercial banks from interstate branching due to concerns that large banking organizations with a presence in several states could not be easily supervised. In the years that followed, bank holding companies were created to circumvent this law and they acquired branches across states, but such practices were terminated with the *Bank Holding Company Act* of 1956.

At the beginning of the 1970s, most states imposed restrictions on geographical expansion both within- and across-state borders, although in many states holding companies could expand by setting up multiple bank subsidiaries.^8^ From the mid-seventies onwards, several states deregulated restrictions on intrastate branching (Jayaratne and Strahan 1996). In this way, they allowed multibank holding companies both to convert their subsidiary banks into branches and to acquire banks and convert them into branches. At the same time, some states lifted restrictions on banks opening new branches within state borders. Although this period was characterized by several waves of mergers,^9^ the US banking system continued to comprise thousands of small, independent banks. According to McCord et al. (2015), in the period 1960–1980, there were between 12 and 13 thousand independent banks.

The creation of an interstate banking market was initiated in 1994 with the approval of the *Interstate Banking and Branching Efficiency Act* (*Riegle-Neal Act*). The Act allowed both unrestricted interstate banking and interstate branching, which meant bank holding companies could acquire banks anywhere in the US and diversify their assets. The reform sought to increase the efficiency of financial institutions, permit the conversion of bank subsidiaries into bank branches and, thus, eliminate indirect costs (McLaughin 1995; Wheelock and Wilson 2004).

Despite the lifting of restrictions, the *Riegle-Neal Act* allowed states to erect certain barriers to protect their banks, and some of them took advantage of these provisions by forbidding out-of-state banks from opening new branches or acquiring existing ones (Johnson and Rice 2008; Rice and Strahan 2010).^10^ A bank could open a new interstate branch if state laws expressly permitted it to do so. Without de novo branching, entry into a particular out-of-state market was only possible via an interstate whole-bank merger. In the years that followed, states individually modified these entry barriers and introduced different measures at different points in time. In 2010, the *Dodd-Frank Act*, section 613, partially reversed state restrictions on de novo branching by out-of-state banks.

In 1999, the *Gramm-Leach-Bliley Act* promoted the integration of the commercial and investment functions of bank entities.^11^ This decision enhanced competition and led to the consolidation of the sector through mergers and acquisitions (Vives 2016). One consequence of these reforms was a reduction in the number of banks, with the smaller, weaker entities being acquired by other institutions seeking to gain economies of scale. In this period, the number of banks continued to decrease, falling to around 10,000 by 2000. Regarding the bank branches, the consolidation of the sector led to a reduction in the number of branches up to 1993, but then there was an important increase, reaching a peak of nearly 100,000 offices in 2009 (FDIC 2012).

After 2007, the sector’s failure to foresee the financial crisis led to the implementation of a series of mechanisms to prevent future problems. The 2010 *Dodd-Frank Wall Street Reform and Consumer Protection Act* sought to protect consumers from abusive financial services practices; empowered the Federal Reserve to supervise the largest, most complex financial companies; established measures to avoid bank bailouts and to monitor risks in the financial system; and prevented banks from becoming “too big to fail”. This generated a series of reforms of the securities market, the regulation of derivatives and the reform of rating agencies (Vives 2016, 2019). In addition, the Act introduced measures that impacted the banking structure, by introducing a degree of separation between the banks’ commercial and investment activities.

**Fig. 1:**
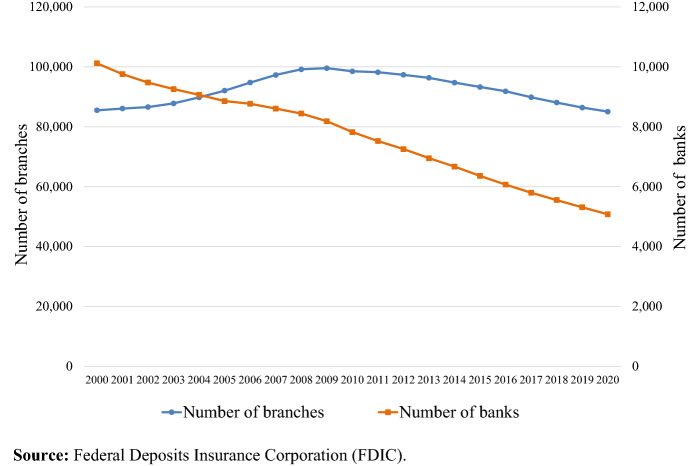
Bank entities and branches, 2000–2020. *Source*: Federal Deposits Insurance Corporation (FDIC)

Figure 1 shows the evolution in the number of banking institutions and branches in the USA from 2000 to 2020. The fall in the total number of banks throughout the period represents a continuation of the trend that began in the 1980s. However, after 2007, this reduction is driven not by the increase in bank failures, but by the lack of bank entry. As McCord et al. (2015) explain, from 2009 through 2013 bank entry fell to almost zero.^12^ Likewise, branch expansion into new geographic markets, which was a notable mechanism for bank entry in previous years, declined dramatically. This can be explained by low bank profitability and the implementation of new banking regulations (Adams and Gramlich 2014). The number of branches, on the other hand, increased until 2009 and then fell after the Great Recession. The de-branching trend of the last decade can be considered a consequence of the consolidation process initiated by the sector after the financial crisis and of the generalized adoption of online banking, associated with cost savings compared to brick-and-mortar branches and a disruptive effect in the traditional retail business. While technological innovations have yet to eliminate the need for branch networks, the frequency of branch use is clearly falling (FDIC 2018).

## Data, trends and methods

### Data

We draw on a dataset for more than 72,738 census tracts located in 3090 counties in the 50 states of the USA and the District of Columbia for the period 2000–2020.^13^ Information on bank branches is obtained from the *Summary of Deposit* (SOD) and *the Reports of Structure Changes* (ROC) provided by the Federal Deposit Insurance Corporation (FDIC). The SOD data are collected for all institutions insured by the FDIC, including commercial banks and savings and loans associations, and they describe the financial situation (deposits and assets) and bank specificities (type of financial services, address, bank affiliation, etc.) at the branch level. The SOD data include details of the branches’ latitude and longitude, allowing us to geo-locate the branches on the map of census tracts using GIS software.

The ROC data describe the non-financial activities of all entities insured by the FDIC, capturing institutional and structural changes including mergers, failures and new offices of financial entities.^14^ As our analysis is conducted at the tract level, we aggregate the local branch information. Specifically, we calculate the branch density, inter- and intrastate mergers, and bank entries and exits for each census tract. We also calculate a market concentration index at the tract level by using information on the banks’ deposits.

Information on the socioeconomic characteristics and median family income at the census tract level has been collected from the US Census Bureau. We use information from the 2000 Decennial Census for the period 2000–2008 and from the American Community Survey (ACS) for the period 2009–2020. The Census also provides the tracts’ land area used to calculate population densities. *Density Population* is the ratio between a tract’s population and its area. *Income* is the median family income in the tract. We also consider the percentage of population over 60 years *(% older*), the percentage of population with a college degree *(% higher education*) and the percentage of population that belongs to minority ethnic groups (*% minority*).

One of the objectives of our analysis is to examine how the emergence of online banking has impacted banks’ de-branching strategy after a merger. To do so, we consider the variable *Broadband Penetration*, which captures the percentage of the population in the tract with a subscription to a fixed broadband Internet connection. Specifically, we use the variable “Residential Fixed High-Speed Connections over 200 kbps in at least one direction per 1000 inhabitants”, which has been obtained from the Federal Communications Commission’s Form 477, available for the period 2009–2018.^15^ This is a categorical variable that shows whether the penetration covers 0–20, 20–40, 40–60, 60–80 or 80–100% of the tract population. In addition, we consider the variable *Fintech Mortgage Lending*, which shows the percentage of mortgage lending covered by FinTech entities at the county level, for the period 2010–2017. This variable only reflects one of the multiple services that might be offered by FinTech entities, but it serves as a good proxy of the relevance of these entities in local markets. This information has been obtained from Fuster et al. (2019), who collected their data from the Home Mortgage Disclosure Act (HMDA) on US mortgage loan originations.^16^

Table 1 presents the descriptive statistics of the variables used in the empirical analysis. Most of the variables show a high degree of variability between tracts both in terms of the standard deviation in relation to the mean and in terms of their minimum and maximum values.

**Table 1:** Descriptive statistics

Variables	Mean	SD	Minimum value	Maximum value
Number of branches per 10,000 in habitants	3.675	6.239	0	807.651
Number of branches per 10,000 in habitants (branches > 0)	5.976	7.037	0.240	807.651
Income (US dollars)	52,379.19	26,281.51	2500	250,000
Density of population (inhabitants per square mile)	4,032.741	10,067.26	0.026	250,850
% older population	0.342	0.115	0.00041	1
% Minority Ethnic Groups	0.211	0.216	0.0001	1
% higher education	0.273	0.179	0.00042	1
HHI deposits	0.737	0.278	0.064	1
Deposits (US dollars)	200,715.6	2,764,323	10,001	5.67e+08
Mergers	0.225	0.715	0	30
Interstate mergers	0.186	0.608	0	25
Intrastate & inter-county mergers	0.032	0.206	0	8
Mergers (> 500 branches)	0.011	0.105	0	1
New Entities	0.0009	0.030	0	2
Saving & Loan Entities	7.813	22.152	0	100
% Lending Fintech	0.054	0.034	0.0007	0.784
Broadband penetration	4.002	1.038	0	5

### Basic trends

Figure 1 shows the evolution in the number of bank entities and branches in the USA in the period 2000–2020. In this period, the number of banks fell from 10,119 to 5076. Until 2008, the number of entities decreased at an average annual rate of 2.2%, accelerating thereafter to an average rate of 4.2%. Despite this process of consolidation, the size of the banks’ branch networks continued to expand until 2009, peaking at 99,550 branches. After that date, the number fell at an average annual rate of 1.3%, down to 85,050 branches in 2020. Notice that between 2008 and 2020 the total population in the USA increased by 8.5%, which means that the average number of inhabitants served by each bank branch increased by 26.5%, from 3067 people per branch in 2008 to 3874 people per branch in 2020 (See Table 9 in the Appendix).^17^

**Fig. 2:**
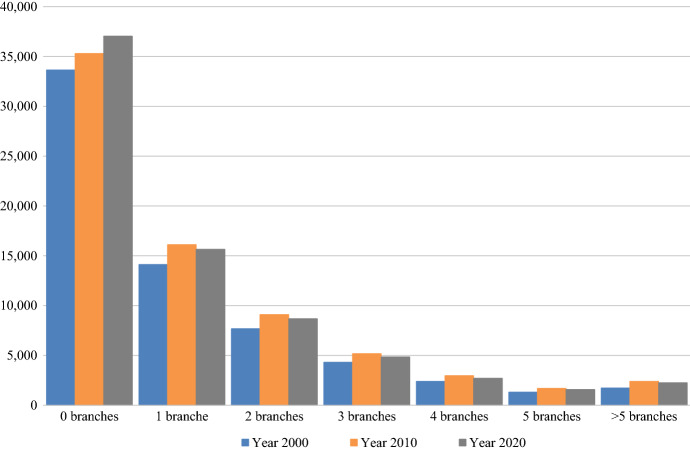
Number of tracts by number of branches. *Note*: The number of tracts changed in the period examined as follows: 65,174 tracts in 2000; 72,714 in 2010; and 72,738 in 2020. *Source*: Federal Deposits Insurance Corporation (FDIC)

The presence of bank branches varies markedly across tracts. Figure 2 shows the distribution of tracts according to the number of branches located in each. The number of tracts defined by the US Census increased in the period examined; according to our dataset, there were 65,174 tracts in 2000, 72,714 in 2010, and 72,738 in 2020. However, the distribution of tracts by number of branches has remained quite stable over time. In 2020, there were 37,044 unbanked tracts (51% of the total), 15,643 tracts with one branch (21.5%), 8676 tracts with two branches (12%), 9119 tracts with three to five branches (12.5%), and 2256 tracts with more than six branches (around 3%). These differences are largely explained by the socioeconomic characteristics of the tracts.

**Fig. 3:**
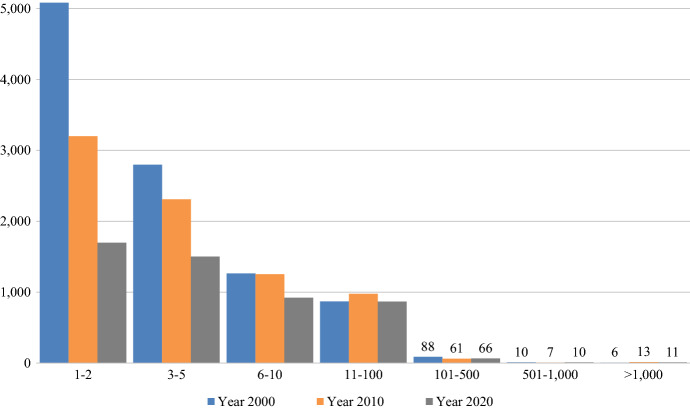
Frequency distribution of bank entities by number of branches. *Note*: The number of bank entities changed in the period examined as follows: 11,119 in 2000; 7821 in 2020; and 5076 in 2020. *Source*: Federal Deposits Insurance Corporation (FDIC)

The number of bank entities has decreased notably in the period 2000–2020, while the number of branches per entity has increased. Figure 3 shows the distribution of banks according to the number of branches they operate and reveals that in the period under study there was a marked concentration in the number of branches. In 2000, of the 10,120 bank entities, around half operated just one or two branches (31% had one branch and 19% had two). In contrast, only six entities had more than 1000 branches: Fleet National Bank (1012 branches), Wells Fargo Bank (1037), U.S. Bank (1075), Sun Trust Bank (1183), Wachovia Bank (2414), and Bank of America (4505). By 2020, the situation had changed dramatically; of the 5076 bank entities, only one-third of these operated one or two branches (19% had one branch and 14% had two). Moreover, there were 11 banks with more than 1000 branches: Citizens Bank (1041), Key Bank (1099 branches), Fifth Third Bank (1137), TD Bank (1226), Regions Bank (1400), PNC Bank (2324), U.S. Bank (2774), Branch Banking and Trust Company (2921), Bank of America (4253), JPMorgan Chase Bank (4979), and Wells Fargo Bank (5410).

**Fig. 4:**
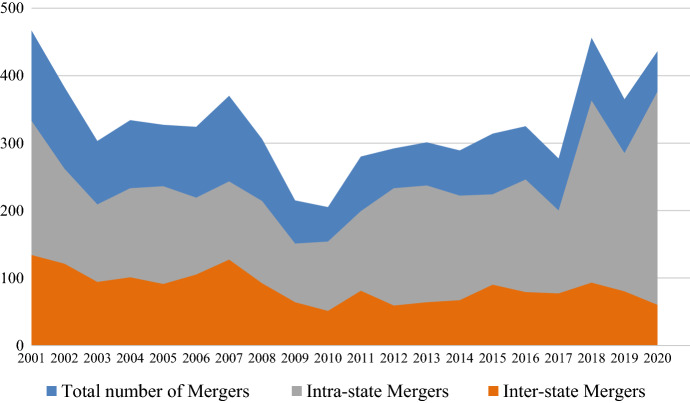
Number of mergers, 2000–2020. *Source*: Federal Deposits Insurance Corporation (FDIC)

One of the factors accounting for this reduction in the number of bank entities and the increase in the concentration of bank branches is the large number of mergers that occurred since 2000. Following the *Riegle Neal Act* of 1994, bank entities engaged in both intra- and interstate mergers aimed at gaining scale and geographically diversifying their risks. After the financial crisis of 2008, banks used mergers to consolidate their position in an environment of weak demand and strong competition. Figure 4 shows the evolution in the number of inter- and intrastate mergers. Over the period under study, at least three of every four mergers were intrastate. The number of interstate mergers decreased from 2000 to 2009 and, following the Great Recession, remained relatively stable. The number of intrastate mergers also fell in the years before 2009, but increased thereafter, especially from 2016 onwards.

### Empirical strategy

The objective of our empirical analysis is to estimate the causal relationship between bank mergers and branch density in the period 2000–2020. According to the literature, two types of effect can be identified because of mergers. First, after the *Riegle-Neal Act*, banks used mergers as a mechanism to enter new markets, reduce their costs and diversify their assets (McLaughin 1995; Cohen and Mazzeo 2010; Aubuchon and Wheelock 2010; Aguirregabiria et al. 2016; Gropp et al. 2019). Considering this, until the Great Recession of 2007–2008, interstate mergers can be expected to have had a positive “expansionary effect” in tracts close to those in which the acquired banks were located. However, these mergers could also have had a negative effect on the branch density of the tracts directly affected by the mergers, as acquirers could have opted to re-structure their branch network or mergers could have deterred the expansion of rival entities.^18^

Second, we expect mergers to have produced a “consolidation effect” in the tracts in which the acquirer and the acquired bank networks overlap. Moreover, we expect this effect to have been stronger after the Great Recession. Following the financial crisis of 2007–2008, the Federal Reserve’s policy of keeping the federal funds rate near zero pushed lending rates down, which kept the net interest margin relatively small. This situation, coupled with the fall in demand (e.g., loans and deposit‐taking services) and the implementation of new banking regulations (e.g., Dodd-Frank Act and FDIC regulations), reduced the profitability of the banks and led to mergers aimed at reducing the costs of premises and improving efficiency (DeYoung et al. 2009).^19^ This consolidation effect, moreover, can be expected to have been stronger in urban tracts, as branch duplications are more likely there. This hypothesis is supported by the literature that points to the relevance of scale economies in banking organizations (McAllister and McManus 1993; Wheelock and Wilson 2001a, b, 2004; Feng and Serilitis 2009). Moreover, urban populations have better access to broadband Internet than those living in rural areas and, therefore, are more likely to bank online.

The main challenge facing our analysis of the causal impact of mergers on the bank market is the potential presence of endogeneity attributable to unobservable factors that might be correlated with branch density and the banks’ merger decisions. To address this potential bias, we take advantage of the panel structure of our dataset to estimate a fixed effects model at the tract level. This allows us to control for the omitted variables that are correlated with the variables of interest, and which do not change over time. Moreover, the model includes county-by-year fixed effects to control for specific idiosyncratic shocks at the county level.

In addition to this empirical strategy, we follow Nguyen (2019) and restrict our analysis to mergers involving banks with at least $1 billion in pre-merger assets and where the acquired bank operated more than 80 branches.^20^ This restriction leaves us with 106 mergers involving 153 large banks in the period 2000–2020. These mergers affected 16,687 tracts belonging to 2127 counties. Mergers between large banks are likely to satisfy several objectives, including expanding to new markets, reducing costs, and increasing market power. However, it is unlikely that such mergers are decided on the basis of the specific socioeconomic conditions of all the local markets affected. To further ensure that this is indeed the case, we exclude from our analysis “intra-county mergers” in which the acquirer and the acquired banks have their headquarters in the same county. As a result, our final sample includes 86 “interstate mergers” between banks from different states and 20 intrastate/inter-county mergers” between banks from the same state but which have their headquarters in different counties.

The variable of interest in our model is *Branch Density*, measured as the number of bank branches per 10,000 inhabitants in tract *k*, in county *c*, and in year *t*. This variable allows us to account for differences in the population size of the tracts. Branch density is determined by local market competition, the banks’ commercial strategy, and the characteristics of the population. Thus, we estimate the following baseline model:

Equation (1):

$${\text{Log}}\;{\text{Branch}}\;{\text{Density}}_{{{\text{kct}}}} = \beta_{1} {\text{Mergers}}_{{{\text{kct}}}} + X_{{{\text{kct}}}}^{\prime } \beta + \delta_{1}^{\prime } {\text{Year}}_{t} + \delta_{2}^{\prime } {\text{Tract}}_{k} + \delta_{3}^{\prime } {\text{Year}}_{t} \cdot {\text{County}}_{c} + \, \varepsilon_{{{\text{kct}}}}$$

where $$X_{{{\text{kct}}}}^{\prime } = \left( {\begin{array}{*{20}l} {{\text{DeNovoBanks}} _{{{\text{kct}}}} , {\text{HHIDeposit}}_{{{\text{kct}} - 1}} ,} \hfill \\ { {\text{Savings}}\& {\text{Loans}}_{{{\text{kct}}}} ,{\text{ LogTotalDeposits}}_{{{\text{kct}} - 1}} ,{\text{LogPopDensity}}_{{{\text{kct}}}} ,} \hfill \\ {{\text{ Log}}\;{\text{Income}}_{{{\text{kct}}}} ,\% {\text{Minority}}_{{{\text{kct}}}} , \% {\text{Higher}}\;{\text{Education}}_{{{\text{kct}}}} , \% {\text{Older}}_{{{\text{kct}}}} } \hfill \\ \end{array} } \right)$$

In this model, the variable *Mergers*_kct_ is the number of times a tract has been exposed to a merger. In one year, tracts may be exposed to as many as three mergers; moreover, each tract can be exposed to several mergers over the entire span of the period. Note that a tract is considered as having been affected by a merger when the acquiring or the acquired bank was operating in the tract before the merger. Additionally, in several specifications of the model, we draw a distinction between interstate mergers and intrastate/inter-county mergers.

The vector $X_{{{\text{kct}}}}^{\prime }$ includes all the control variables considered in the model. First, we consider a group of variables related to the level of competition in the market. De Novo* Banks* reflects the entry of new banks in the tract, which can have positive effects on the number of branches. This variable takes a value equal to 1 when a bank starts operating within the tract. *HHI Deposits* is a Herfindahl–Hirschman index (HHI) that measures the concentration of bank entities’ deposits at the tract level. We expect tracts with a high market concentration to have fewer bank branches. To account for the possible endogeneity of this variable, we include it in the model with a one-year lag. *Savings & Loans* is a variable that reflects the percentage of Savings and Loan institutions in the tract. These are banks that a priori are locally oriented and place stronger emphasis on residential mortgages, while commercial banks tend to concentrate more on businesses and unsecured credit services such as credit cards. Hence, we expect Savings & Loan banks to have more branches than their commercial counterparts. We also consider the *Total Deposits* kept by residents in the tract branches. The existence of a large volume of deposits in the tract should induce banks to create new branches to compete more effectively against rivals. The potential endogeneity of this variable is treated by including it with a lag.^21^

Equation (1) also considers a set of socioeconomic variables that reflect the characteristics of the tracts, namely population density (*Population Density),* median income *(Income),* percentage of population represented by minority ethnic groups (*% Minority),* percentage of population with a college degree (*% Higher Education),* and percentage of population over the age of 60 *(% Older)*.

**Fig. 5:**
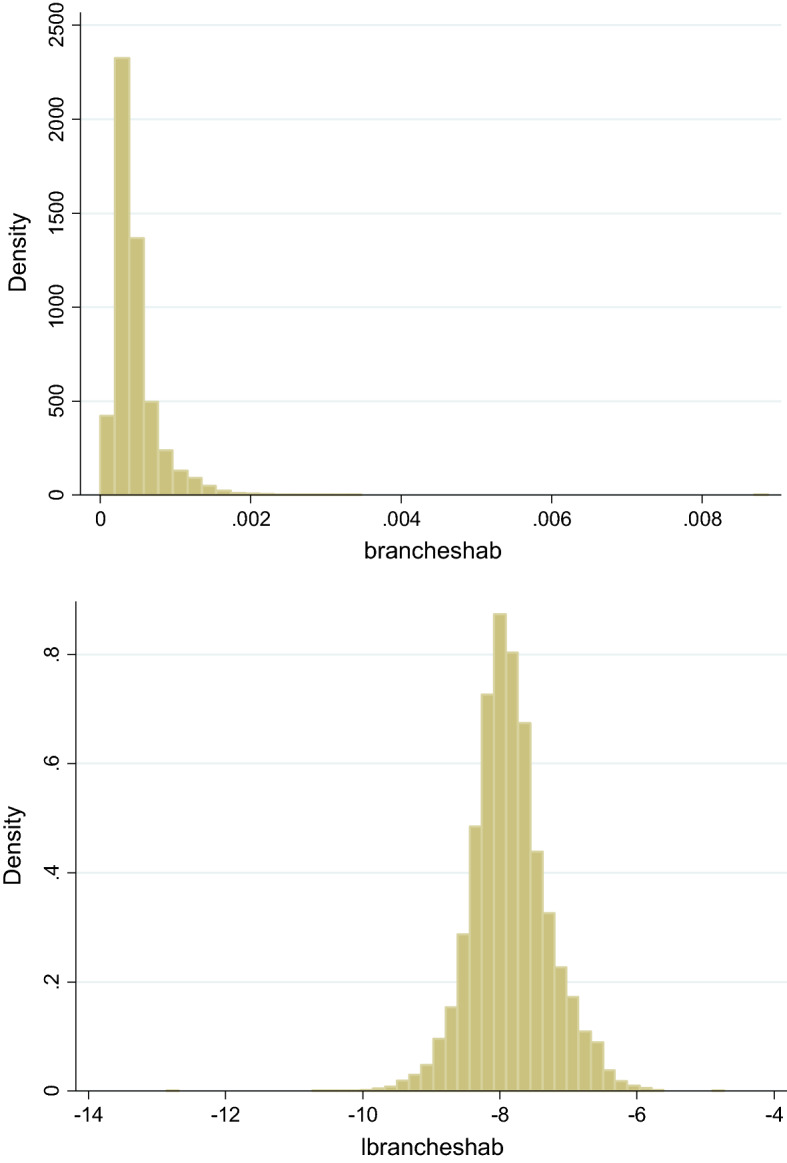
Histograms of the dependent variable

Note that all variables without bounded values are transformed in logarithms. The log-transformation of the variables allows us to address the severe skewness that might affect some of them, particularly the dependent variable. It also allows us to reduce the influence of outliers and to interpret the coefficients in terms of elasticities. The use of logged values of the dependent variable implies that our analysis is restricted to tracts that have at least one bank branch, which provides us with a better comparable sample of tracts both exposed and unexposed to mergers. Figure 5 in the Appendix shows that the distribution of the dependent variable without logs is severely skewed towards zero, while it is much smoother when we use logged values.

Finally, the model in Eq. (1) also includes year-fixed effects (*Year*_*t*_), tract-fixed effects (*Tract*_*k*_) and the interaction of county and year-fixed effects (*Year*_*t*_ ·County_*c*_).

The estimation of the model may present problems of heteroscedasticity and temporal autocorrelation in the error term. In all regressions, the Breusch–Pagan/Cook–Weisberg test shows heteroscedasticity problems so that standard errors are robust to heteroscedasticity. Likewise, the Wooldridge test for autocorrelation in panel data reveals that a problem of serial autocorrelation may exist so that we use standard errors clustered by tract (Bertrand et al. 2004).

## Results

Below, we present the results of the fixed effects model examining the impact of mergers between large, multi-branch banks on branch density at the tract level. Table 2 summarizes the results of our analysis for the whole period 2000–2020. Columns 1 and 2 show the estimates when using the sample containing all the tracts with bank branches *in counties* that were exposed to the merger of a large bank in the period under study. Column 1 reports the effect of all mergers on branch density, and column 2 shows the separate effects for interstate mergers and intrastate/inter-county mergers. Mergers reduced branch density in the exposed tracts by 2.8% in comparison with unexposed tracts. Our results for interstate mergers are like those found for all merger types. Tracts affected by intrastate/inter-county mergers presented a 4.5% reduction in branch density in comparison with untreated tracts. Columns 3 and 4 repeat the analysis, but the sample is now restricted to *tracts* that during the period under study were exposed to the merger of a large bank. This makes the group of tracts exposed and unexposed to a merger more comparable. The results for the impact of mergers on branch density are unchanged, confirming the conclusion that mergers, especially intrastate/inter-county mergers, had a negative impact on branch density.

**Table 2:** Impacts of mergers on branch density

Variables	(1)	(2)	(3)	(4)
Mergers	− 0.0282***		− 0.0292***	
	(0.00165)		(0.00188)	
Interstate mergers		− 0.0274***		− 0.0279***
		(0.00180)		(0.00201)
Intrastate/inter-county mergers		− 0.0453***		− 0.0465***
		(0.00541)		(0.00534)
Lag total deposits	0.0455***	0.0455***	0.0685***	0.0685***
	(0.000949)	(0.000948)	(0.00181)	(0.00181)
Lag HHI deposits	− 0.827***	− 0.827***	− 0.857***	− 0.858***
	(0.00482)	(0.00482)	(0.00730)	(0.00731)
Saving & loan entities	0.000954***	0.000958***	0.00124***	0.00125***
	(6.99e−05)	(6.99e−05)	(0.000106)	(0.000106)
Income	0.0187**	0.0185**	0.0262**	0.0255**
	(0.00757)	(0.00756)	(0.0115)	(0.0114)
Density of population	− 0.838***	− 0.838***	− 0.864***	− 0.864***
	(0.0119)	(0.0119)	(0.0166)	(0.0166)
New entities	0.149***	0.149***	0.143***	0.144***
	(0.0120)	(0.0120)	(0.0144)	(0.0144)
% Minority	0.0301*	0.0288*	0.0508**	0.0485*
	(0.0169)	(0.0169)	(0.0252)	(0.0252)
% higher education	0.128***	0.129***	0.160***	0.162***
	(0.0226)	(0.0226)	(0.0346)	(0.0346)
% older	0.108***	0.109***	0.125***	0.126***
	(0.0191)	(0.0191)	(0.0286)	(0.0286)
Constant	6.683***	6.732***	8.814***	8.976***
	(0.426)	(0.425)	(0.690)	(0.684)
Observations	610,186	610,186	263,365	263,365
*R*^2^	0.269	0.269	0.310	0.310
Number of tracts	49,698	49,698	16,687	16,687
Tract FE	Yes	Yes	Yes	Yes
Year FE	Yes	Yes	Yes	Yes
County × Year FE	Yes	Yes	Yes	Yes
Sample	Treated counties	Treated counties	Treated tracts	Treated tracts
Period	All	All	All	All

Robust standard errors in parentheses and clustered at the tract level

***p < 0.01, **p < 0.05, *p < 0.1. All continuous variables are in logs

The results for the control variables in Table 2 are in line with expectations. We find that branch density is positively associated with total deposits and income in the tract and negatively associated with market concentration measured with the HHI index. Moreover, bank entry and the presence of Savings & Loan entities are associated with a higher branch density. As for our sociodemographic indicators, we find that the percentage of population with a higher education, the percentage of population belonging to minority ethnic groups and the percentage of older population are positively associated with a high number of branches. In contrast, density of population is negatively associated with branch density.^22^

### Impact of the Great Recession

One of the hypotheses of our study is that after the *Riegle-Neal Act* of 1994 banks used mergers as a mechanism to expand their activities to other states and that this led to an increase in branch density. This expansion effect could have been reversed after the 2007 financial crisis, as banks tried to reduce their costs by closing branches in less profitable local markets. To analyze the existence of this differential impact of mergers before and after the crisis, Table 3 divides the sample in two periods. Columns 1 and 2 show the results for the years before the Great Recession (2000–2007) and Columns 3 and 4 for the years after (2008–2020). We find very similar outcomes for the two periods, albeit the impact is stronger after the crisis. Before the crisis, direct exposure to a merger implied a 2.6% reduction in branch density in the affected tracts, while after the crisis the implied reduction was 3.2%. Moreover, while interstate mergers had similar effects in the two periods, intrastate/inter-county mergers had a stronger impact on branch density after the crisis. Table 10 in the Appendix repeats the analysis though this time dividing the sample in four periods. The results are essentially the same; if anything, we observe an intensification of the effect in exposed tracts after the crisis.

**Table 3:** Impact of mergers on branch density: Pre- and Post-Crisis Effects

Variables	(1)	(2)	(3)	(4)
Mergers	− 0.0263***		− 0.0323***	
	(0.00187)		(0.00329)	
Interstate mergers		− 0.0247***		− 0.0311***
		(0.00207)		(0.00355)
Intrastate/inter-county mergers		− 0.0287***		− 0.0439***
		(0.00483)		(0.0116)
Constant	− 18.36***	− 17.40***	39.20***	39.21***
	(1.096)	(1.083)	(3.673)	(3.673)
Observations	109,225	109,225	154,140	154,140
*R*^2^	0.255	0.255	0.193	0.193
Number of tracts	14,638	14,638	16,248	16,248
Tract FE	Yes	Yes	Yes	Yes
Year FE	Yes	Yes	Yes	Yes
County × Year FE	Yes	Yes	Yes	Yes
Sample	Treated tracts	Treated tracts	Treated tracts	Treated tracts
Period	Pre-crisis	Pre-crisis	Post-crisis	Post-crisis

Robust standard errors in parentheses and clustered at the tract level

***p < 0.01, **p < 0.05, *p < 0.1. All continuous variables are in logs

Another interesting analysis is to consider the effect of mergers for very large banks. Table 11 in the Appendix repeats the previous analysis but focuses specifically on mergers in which the acquired bank had more than 500 branches at the time of the transaction. In this instance, we do not find a negative impact on exposed tracts before the crisis, and we observe a 1.4% reduction in branch density after the crisis.

Table 4 verifies the robustness of these results by replicating the analysis with a matched sample. Our previous analysis focused on tracts exposed to the merger of a large bank, but it did not consider the existence of preexisting observable differences between tracts exposed and unexposed to mergers that might impact our results. Indeed, tracts exposed to a merger might differ in several respects to unexposed tracts in the same county. To address this possibility, we apply a propensity score matching procedure to build a subsample of unexposed tracts that are similar in terms of their observable factors to those exposed to the mergers. Matching techniques eliminate possible selection biases by pairing the tracts affected by mergers with those unaffected by such transactions and allow us to re-estimate the baseline model with the observations that have common support (Shen et al. 2016, 2020). The variables included in our matching analysis are the socioeconomic characteristics of the tracts (*Population Density*, *% Older*, *% Minority*, *% Higher Education*, *Income*). Specifically, we impose that the tracts exposed and unexposed to the mergers are similar in terms of these characteristics in all the years of the analysis. Moreover, our analysis considers all tracts in counties affected by mergers in the period examined. Table 12 in the Appendix reports the differences in means between treated counties (counties affected by mergers) and control counties (counties unaffected by mergers) when considering both the full sample and the matched sample. The table shows that the differences between treated and control tracts are smaller in the case of the matched sample than in that of the full sample, although some significant differences are still present.

**Table 4:** Impact of mergers on branch density: Matching Sample

Variables	(1)	(2)	(3)	(4)	(5)	(6)
Mergers	− 0.0294***		− 0.0230***		− 0.0360***	
	(0.00177)		(0.00176)		(0.00317)	
Interstate mergers		− 0.0283***		− 0.0219***		− 0.0353***
		(0.00191)		(0.00195)		(0.00344)
Intrastate/inter-county mergers		− 0.0468***		− 0.0248***		− 0.0428***
		(0.00538)		(0.00474)		(0.0116)
Constant	7.196***	7.316***	− 15.69***	− 15.12***	39.60***	39.59***
	(0.581)	(0.578)	(0.872)	(0.864)	(3.536)	(3.537)
Observations	375,059	375,059	152,044	152,044	221,350	221,350
*R*^2^	0.282	0.282	0.251	0.251	0.177	0.177
Number of tracts	42,889	42,889	22,359	22,359	40,229	40,229
Tract FE	Yes	Yes	Yes	Yes	Yes	Yes
Year FE	Yes	Yes	Yes	Yes	Yes	Yes
County × Year FE	Yes	Yes	Yes	Yes	Yes	Yes
Sample	Matching & Treated counties	Matching & Treated counties	Matching & Treated counties	Matching & Treated counties	Matching & Treated counties	Matching & Treated counties
Period	All	All	Pre-crisis	Pre-crisis	Post-crisis	Post-crisis

Robust standard errors in parentheses and clustered at the tract level

***p < 0.01, **p < 0.05, *p < 0.1. All continuous variables are in logs

Table 4 presents the results of the matching analysis for the whole period and for the periods before and after the Great Recession. The results obtained are very similar to those in Table 3, confirming that in the pre- and post-crisis periods, local markets responded to the mergers with a similar adjustment in their branch density. We also obtain similar results for the two merger types, with stronger negative effects after the crisis, especially for intrastate mergers.

### Effects at the county level

To evaluate the importance of mergers as a mechanism enabling banks to expand their activities to other markets, we next analyze the effects of mergers at the county level. Column 1 in Table 5 considers the effects of mergers for the whole 2000–2020 period and shows that they had a negative, although small, impact on branch density. Column 4 examines the separate effect of interstate mergers and intrastate/inter-county mergers: in the case of the former, each merger increased branch density by 0.2%, while in the case of the latter, each merger reduced this density by 1.2%. If we focus on the pre-crisis period, we find that interstate mergers increased branch density by 0.5%, while intrastate mergers reduced it by 1.6%, while, in the post-crisis period, each inter- and intrastate merger reduced branch density at the county level, by 1.4 and 1.9%, respectively.

**Table 5:** Impact of large mergers of branch density: county level

Variables	(1)	(2)	(3)	(4)	(5)	(6)
Mergers	− 0.000932*	− 0.000444	− 0.0143***			
	(0.000558)	(0.00158)	(0.00173)			
Interstate mergers				0.00202**	0.00521**	− 0.0149***
				(0.000797)	(0.00207)	(0.00220)
Intrastate/inter-county mergers				− 0.0120***	− 0.0164***	− 0.0190***
				(0.00191)	(0.00351)	(0.00388)
Constant	0.801***	− 1.896***	2.587***	0.792***	− 1.885***	2.591***
	(0.131)	(0.216)	(0.293)	(0.131)	(0.215)	(0.293)
Observations	64,544	24,602	39,942	64,544	24,602	39,942
*R*^2^	0.216	0.261	0.189	0.216	0.263	0.189
Number of counties	3127	3093	3125	3127	3093	3125
Year FE	Yes	Yes	Yes	Yes	Yes	Yes
County FE	Yes	Yes	Yes	Yes	Yes	Yes
State × Year FE	Yes	Yes	Yes	Yes	Yes	Yes
Period	All	Pre-crisis	Post-crisis	All	Pre-crisis	Post-crisis

Robust standard errors in parentheses and clustered at the county level

***p < 0.01, **p < 0.05, *p < 0.1. All continuous variables are in logs

To sum up, these results imply that before the crisis *tracts* directly exposed to *interstate mergers* increased their branch density at a smaller rate than tracts unexposed to such mergers. This might be attributable to strategies of consolidation or could reflect the deterrence effect of mergers on rival banks. However, at the *county level* these mergers generated an increase in branch density. After the Great Recession, this expansionary effect was no longer present as both tracts and counties were negatively affected by interstate mergers. In the case of *intrastate mergers* between large banks, no evidence is found of an expansionary effect at the *county level* before the crisis. Indeed, both tracts and counties exposed to these mergers lost branch density with respect to unexposed tracts. However, after the crisis, we find that intrastate/inter-county mergers had a stronger impact.

### Effects in tracts with overlapping branch networks

To further evaluate the effects of mergers, we next consider those cases in which prior to the transaction the acquirer and the acquired banks had overlapping retail branch networks in the same tract. We expect that in tracts with a duplicate presence the merged entity will be more likely to close branches. Table 6 examines the effects of mergers in tracts with and without overlapping networks. Column 1 considers the effects of all mergers and shows that in tracts without a duplicate presence, mergers reduced branch density by 0.6% while in tracts with overlapping networks density fell by 6.4%. Columns 2 and 3 consider this effect before and after the financial crisis, respectively, and show that the consolidation effect in tracts with overlapping branch networks was 8.0 and 8.1%. Columns 4–6 replicate the previous analysis but differentiate between inter- and intrastate mergers. We find that the consolidation effect of interstate mergers in tracts with a duplicate presence was 6.0% before the crisis and 5.5% after the crisis. In tracts without overlapping branch networks, mergers only had a significant negative effect before the crisis. The impact of intrastate mergers in tracts with a duplicate presence was a 5.1% reduction in branch density before the crisis and a 12.6% reduction after the crisis. In tracts without overlapping branch networks, intrastate mergers reduced branch density by 2.0% before the crisis and by 3.5% after the crisis.

**Table 6:** Mergers effects in tracts with overlapping branches

Variables	(1)	(2)	(3)	(4)	(5)	(6)
Merger × No overlap	− 0.00683**	− 0.0290***	− 0.00829			
	(0.00307)	(0.00286)	(0.00574)			
Merger × overlap	− 0.0642***	− 0.0805***	− 0.0814***			
	(0.00826)	(0.00910)	(0.0138)			
Interstate mergers × no overlap				− 0.0124***	− 0.0338***	− 0.00980
				(0.00315)	(0.00317)	(0.00643)
Interstate mergers × overlap				− 0.0389***	− 0.0609***	− 0.0550***
				(0.00827)	(0.0110)	(0.0144)
Intrastate mergers × no overlap				− 0.0278***	− 0.0203***	− 0.0351***
				(0.00674)	(0.00622)	(0.0134)
Intrastate mergers × overlap				− 0.0779***	− 0.0518***	− 0.126***
				(0.0150)	(0.0129)	(0.0322)
Constant	13.26***	− 15.70***	39.84***	12.48***	− 17.63***	40.02***
	(0.675)	(1.044)	(3.671)	(0.683)	(1.158)	(3.642)
Observations	263,365	109,225	154,140	263,365	95,423	140,975
*R*^2^	0.308	0.254	0.192	0.309	0.224	0.194
Number of tracts	16,687	14,638	16,248	16,687	14,555	16,187
Tract FE	Yes	Yes	Yes	Yes	Yes	Yes
Year FE	Yes	Yes	Yes	Yes	Yes	Yes
County × Year FE	Yes	Yes	Yes	Yes	Yes	Yes
Sample	Treated tracts	Treated tracts	Treated tracts	Treated tracts	Treated tracts	Treated tracts
Period	All	Pre-crisis	Post-crisis	All	Pre-crisis	Post-crisis

Robust standard errors in parentheses and clustered at the tract level. All continuous variables are in logs

***p < 0.01, **p < 0.05, *p < 0.1

These results confirm the hypothesis that the most intense branch consolidation effect took place following mergers in tracts in which banks had an overlapping presence before the transaction. The impact was particularly high in the case of intrastate mergers after the crisis. However, we also find a significant, albeit more modest, impact of mergers in tracts without overlapping retail branch networks. Finally, it is worth stressing that our results here are in line with Nguyen (2019), who studied the effect of 13 large mergers in the period 1999–2012 and found that mergers in tracts with overlapping branch networks increased the probability of branch closure by 27%.

### Mergers and online banking

In the last decade, the digitalization of the economy has allowed banks to offer many of their services online and this has favored the development of FinTech services. The rapid expansion of online banking has substantially reduced the number of customer visits to brick-and-mortar branches and has led banks to close their less profitable branches. Below, we re-estimate Eq. (1) to determine the impact of the adoption of fixed broadband Internet and FinTech services—which has been quite heterogeneous across the US tracts—on banks’ decision to close branches after a merger.

FinTech are institutions that use new applications, processes, and business models offered through the Internet to provide financial services to consumers. As explained in Sect. 3.1, we use the variable *Lending FinTech*, as devised by Fuster et al. (2019), as a proxy of the adoption of FinTech services. This is a continuous variable that shows the percentage of mortgage lending covered by FinTech entities at the county level for the period 2010–2017. Columns 1 and 2 in Table 7 analyze the impact of mergers on branch density at the tract level after controlling for FinTech adoption. Column 1 shows that the penetration of FinTech services is positively related to branch density. Column 2 includes the interaction of the *Mergers* and *Lending FinTech* variables. In this case, the variable *Lending FinTech* is no longer significant, and the interaction of the variables presents a negative sign that is also not significant. Our interpretation of this result is that for the period analyzed mortgage lending by FinTech entities remained relatively small and differences in its adoption across tracts was not sufficiently relevant to influence the banks’ decisions.

**Table 7:** Mergers effects and online banking

Variables	(1)	(2)	(3)	(4)	(5)	(6)
Mergers	− 0.0458***	− 0.0457***	− 0.0456***	0.00840	0.00918	0.0434
	(0.00558)	(0.00557)	(0.00460)	(0.00783)	(0.00845)	(0.0337)
Lending fintech	1.62e−10**	3.64e−10				
	(6.43e−11)	(3.05e−10)				
Broadband			0.000439	0.0196***	0.0207***	0.0308***
			(0.00253)	(0.00354)	(− 0.00379)	(0.0126)
Mergers × lending fintech		− 1.68e−10				
		(2.71e−10)				
Mergers × broadband				− 0.0126***	− 0.0126***	− 0.0231***
				(0.00156)	(0.00164)	(0.00976)
Constant	9.201***	9.199***	8.497***	8.375***	40.85***	45.86***
	(0.204)	(0.204)	(0.172)	(0.173)	(3.399)	(13.60)
Observations	92,671	92,671	117,774	117,774	108,540	9,234
*R*^2^	0.113	0.113	0.166	0.167	0.175	0.144
Number of tracts	13,336	13,336	16,163	16,163	14,869	1492
Tract FE	Yes	Yes	Yes	Yes	Yes	Yes
Year FE	Yes	Yes	Yes	Yes	Yes	Yes
County × Year FE	Yes	Yes	Yes	Yes	Yes	Yes
Sample	Treated tracts	Treated tracts	Treated tracts	Treated tracts	Urban & treated tracts	Rural & treated tracts
Period	2010–2017	2010–2017	2009–2018	2009–2018	2009–2018	2009–2018

Robust standard errors in parentheses and clustered at the tract level. All continuous variables are in logs

***p < 0.01, **p < 0.05, *p < 0.1

In an alternative approach aimed at accounting for the digitalization of the bank market, we opted to estimate the effects of mergers using the variable *Broadband Penetration* as a control. As explained, this variable comprises five categories that reflect the percentage uptake of fixed broadband Internet subscriptions in each tract. Column 3 in Table 7 shows that the penetration of broadband Internet has not had a significant impact on branch density. Column 4 includes the interaction of the *Mergers* and *Broadband Penetration* variables. In this case, we find that *Broadband Penetration* is positively related to branch density. Moreover, the coefficient of the interaction between the two variables is negative and significant, which implies that after a merger banks were more likely to close their branches in tracts in which there was a high penetration of broadband Internet. This occurred both in urban and rural tracts, even though broadband penetration is higher in urban tracts. This outcome suggests that the Internet channel is a substitute for many activities of brick-and-mortar branches and that the deployment of high-capacity telecommunications networks can accelerate the closure of bank branches. Notice, also, that branch closures might have notable consequences for consumers, especially if banks do not compensate for such a policy by increasing their online attention to consumers, and if a sizeable proportion of the population in the local markets do not have good access to broadband services and digital skills.

Finally, we should note that the variables *Lending FinTech* and *Broadband Penetration* present two limitations. First, data are unavailable for the last few years of the period we examine, which are precisely the years in which online banking services have undergone their greatest expansion. And second, the FinTech variable only considers one specific service and not all the activities generated by these entities. Despite these limitations, our results for broadband penetration suggest that differences in the effects of mergers before and after the recession could have been exacerbated by the boom in online banking and FinTech.

### Differences between urban and rural populations

This section explores the heterogeneous effects of bank mergers across urban and rural populations. Data from the American Community Survey (ACS) indicate that in 2018 about 19% of the US population lived in rural areas, representing as much as 97% of the total land area. The delineation between urban and rural populations is based essentially on population density.^23^ In our analysis, we classify tracts as urban when more than 50% of the population lives in an urban area. Note that our results are very similar when we define tracts as urban when more than 75% of the population lives in an urban area, albeit that the number of rural tracts decreases notably. Based on this definition, our sample includes 15,343 urban tracts and 1568 rural tracts affected by mergers. Notice that, in more recent years, large banks have tended to focus their commercial activity in urban markets and ceded business to smaller banks and community banks in slower-growing and less-profitable rural markets. As a result, small banks have not lost their position in rural areas.^24^

Panel A in Table 8 presents the estimates of our analysis for urban tracts. We find that for the whole period from 2000 to 2020 mergers reduced branch density by around 2.9%, the impact being slightly greater after the financial crisis. We do not find any specific differences between the impact of inter- and intrastate mergers, particularly when we consider the periods before and after the crisis.

**Table 8:** Impact of mergers on branch density: ﻿Urban vs Rural tracks

Variables	(1)	(2)	(3)	(4)	(5)	(6)
Period	All	Pre-crisis	Post-crisis	All	Pre-crisis	Post-crisis
*Panel A: Urban tracts*						
Mergers	− 0.0294***	− 0.0262***	− 0.0322***			
	(0.00195)	(0.00194)	(0.00344)			
Interstate mergers				− 0.0288***	− 0.0246***	− 0.0328***
				(0.00209)	(0.00215)	(0.00370)
Intrastate/inter-county mergers				− 0.0413***	− 0.0262***	− 0.0276**
				(0.00562)	(0.00501)	(0.0120)
Constant	8.548***	− 18.57***	37.93***	8.730***	− 17.50***	37.91***
	(0.720)	(1.161)	(3.842)	(0.714)	(1.146)	(3.842)
Observations	241,645	99,672	141,973	241,645	99,672	141,973
*R*^2^	0.313	0.253	0.195	0.313	0.252	0.195
Number of tracts	15,343	13,324	14,947	15,343	13,324	14,947
Tract FE	Yes	Yes	Yes	Yes	Yes	Yes
Year FE	Yes	Yes	Yes	Yes	Yes	Yes
County × Year FE	Yes	Yes	Yes	Yes	Yes	Yes
Sample	Urban & treated tracts	Urban & treated tracts	Urban & treated tracts	Urban & treated tracts	Urban & treated tracts	Urban & treated tracts
Banks	Big	Big	Big	Big	Big	Big
Period	All	Pre-crisis	Post-crisis	All	Pre-crisis	Post-crisis
*Panel B: Rural tracts*						
Mergers	− 0.0199***	− 0.0223***	− 0.0216*			
	(0.00705)	(0.00663)	(0.0124)			
Inter states mergers				− 0.00709	− 0.0199***	0.00347
				(0.00767)	(0.00716)	(0.0136)
Intra state/inter county mergers				− 0.0865***	− 0.0521***	− 0.140***
				(0.0169)	(0.0178)	(0.0359)
Constant	12.35***	− 16.20***	60.42***	12.74***	− 16.61***	61.88***
	(2.400)	(3.174)	(12.35)	(2.370)	(3.169)	(12.33)
Observations	21,720	9553	12,167	21,720	9553	12,167
*R*^2^	0.263	0.318	0.178	0.265	0.318	0.181
Number of incode	1568	1314	1506	1568	1314	1506
Tract FE	Yes	Yes	Yes	Yes	Yes	Yes
Year FE	Yes	Yes	Yes	Yes	Yes	Yes
County × Year FE	Yes	Yes	Yes	Yes	Yes	Yes
Sample	Rural & treated tracts	Rural & treated tracts	Rural & treated tracts	Rural & treated tracts	Rural & treated tracts	Rural & treated tracts
Banks	Big	Big	Big	Big	Big	Big

Robust standard errors in parentheses and clustered at the tract level. All continuous variables are in logs

***p < 0.01, **p < 0.05, *p < 0.1

Panel B presents our estimates for rural tracts. Our results show that mergers reduced branch density by around 2%, the impact being similar before and after the financial crisis. Interstate mergers of large banks only had a negative effect on branch density before the crisis but did not affect the number of branches after the crisis. In contrast, intrastate mergers reduced branch density by 8.6%. Before the crisis, these mergers reduced branch density by 5.2%, while after the crisis the negative effect increased to 14%.

## Conclusions

At the end of the 1990s, the deregulation of interstate banking and branching was seen as an opportunity for many large banks to expand their activities to other states. In a period of population growth and high demand for financial services, banks used mergers to enter new markets, as these allowed them to gain in scale, implement new technologies and management practices, and diversify their risk. Our analysis has shown that interstate mergers increased the number of bank branches in the counties affected by this expansion strategy. However, the tracts directly exposed to the mergers experienced a smaller increase in their branch density than that experienced by unexposed tracts. In the case of intrastate mergers, both the counties and the tracts directly exposed to the mergers experienced a smaller increase in their branch density than was experienced by unexposed counties and tracts. One explanation for this consolidation effect is the closure of branches in tracts in which the acquirer and the acquired entities had a duplicate commercial presence, yet branch closures also took place in tracts without overlapping branch networks. This suggests that mergers were an opportunity for banks to restructure their retail networks, and in addition mergers could deter entry in local markets.

Following the 2007–2008 financial crisis, the decrease in the banks’ profitability due to the fall in demand and low interest margins led to branch closures, which helped the sector reduce its costs. The average number of inhabitants served per branch rose from 3067 in 2009 to 3874 in 2020, representing an increase of 26.5%. In this period, there was a marked growth in the number of mergers, which served to foster market concentration. Our analysis has shown that in these years both inter- and intrastate mergers of large banks had a negative effect on branch density at the tract and county levels. The effects of intrastate mergers were especially strong in rural tracts and in tracts in which merging banks had overlapping branch networks.

The banks’ efficiency targets changed after the crisis, triggered, in all probability, by the desire to divert more customers to their online services. In recent years, the coronavirus pandemic has served to reinforce this trend. Clients have quickly become accustomed to online banking, and this change in consumer behavior has led banks to reduce their branch operations. One contribution of our paper has been to analyze the way in which banks have adjusted their de-branching strategy in the wake of a merger taking into consideration the level of penetration of broadband Internet and FinTech services. We have shown that, following a merger, banks have closed more branches in tracts with a relatively higher penetration of broadband Internet services; however, we do not find any evidence that mortgage lending by FinTech entities has modified the banks’ branch networks.

The main implications of our findings are that public policies promoting bank competition and online banking need to consider their impact on financial exclusion. There is an extensive literature showing that the intensification of competition promotes access to banking services for low-income households, yet market consolidation reduces branch density and can negatively affect the access of the more vulnerable population to financial services. Moreover, branch closures reduce soft information and are associated with an increase in consumer bankruptcy. Improving Internet access in less-dense areas and offering financial advice to vulnerable households seem necessary measures to mitigate the progressive reduction in bank branch networks.

## Figures and Tables

**Fig. 6:**
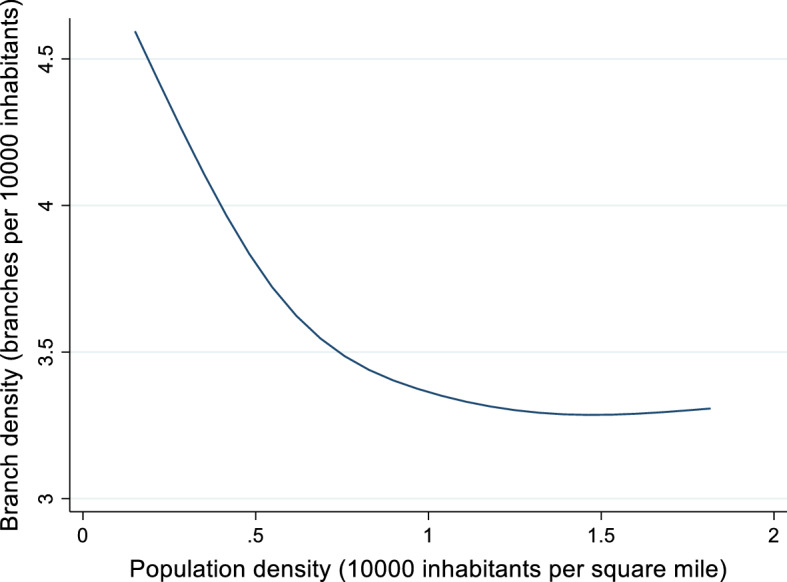
Branch density and population density: urban tracts

**Fig. 7:**
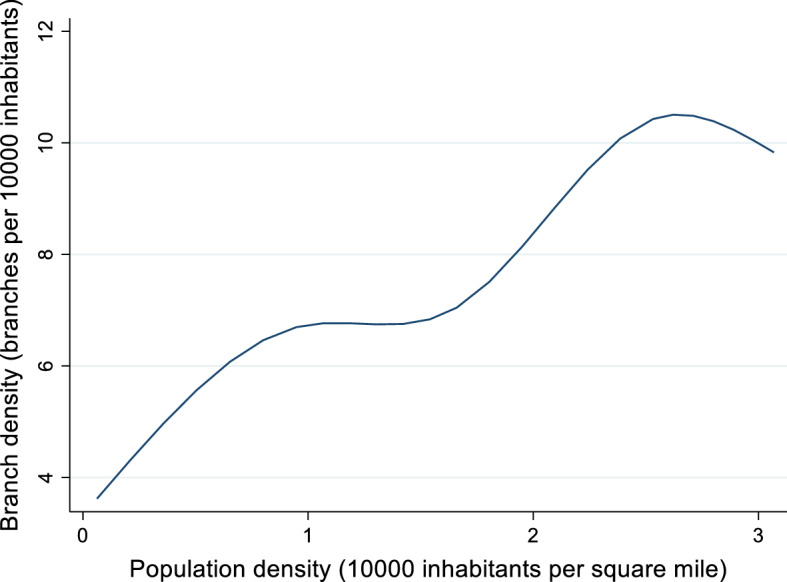
Branch density and population density: rural tracts

## Appendix

**Table 9:** Evolution of bank branches and population, 2000–2020. *Source*: FDIC and US Census Bureau

Year	Population	Variation population (%)	Branches	Variation branches (%)	Population/branches	VariationPop/branches (%)
2000	282,162,411	–	85,492	–	3300	–
2001	284,968,955	0.99	86,069	0.67	3311	0.66
2002	287,625,193	0.94	86,577	0.59	3322	0.34
2003	290,107,933	0.86	87,785	1.40	3305	− 0.52
2004	292,805,298	0.93	89,785	2.28	3261	− 1.32
2005	295,516,599	0.92	92,043	2.51	3211	− 1.55
2006	298,379,912	0.94	94,752	2.94	3149	− 1.92
2007	301,231,207	0.97	97,274	2.66	3097	− 1.66
2008	304,093,966	0.92	99,163	1.94	3067	− 0.97
2009	306,771,529	0.88	99,550	0.39	3082	0.49
2010	309,327,143	0.84	98,519	− 1.04	3140	1.89
2011	311,583,481	0.71	98,193	− 0.33	3173	1.06
2012	313,877,662	0.74	97,340	− 0.87	3225	1.62
2013	316,059,947	0.70	96,339	− 1.03	3281	1.74
2014	318,386,329	0.72	94,725	− 1.68	3361	2.45
2015	320,738,994	0.75	93,272	− 1.53	3439	2.31
2016	323,071,755	0.71	91,834	− 1.54	3518	2.30
2017	325,122,128	0.65	89,849	− 2.16	3619	2.86
2018	326,838,199	0.52	88,075	− 1.97	3711	2.55
2019	328,329,953	0.45	86,392	− 1.91	3800	2.41
2020	329,484,123	0.33	85,050	− 1.55	3874	1.93

**Table 10:** Impact of mergers on branch density: Short intervals

Variables	(1)	(2)	(3)	(4)
Mergers	− 0.0337***	− 0.0350***	− 0.0352***	− 0.0408***
	(0.00211)	(0.00337)	(0.00788)	(0.00551)
Constant	− 19.98***	16.55	25.84***	42.74***
	(1.393)	(12.84)	(6.895)	(2.904)
Observations	81,638	64,508	58,880	58,339
*R*^2^	0.184	0.076	0.112	0.119
Number of tracts	14,437	14,379	12,780	12,503
Tract FE	Yes	Yes	Yes	Yes
Year FE	Yes	Yes	Yes	Yes
County × Year FE	Yes	Yes	Yes	Yes
Sample	Treated tracts	Treated tracts	Treated tracts	Treated tracts
Period	2000–2005	2006–2010	2011–2015	2016–2020

Robust standard errors in parentheses and clustered at the tract level

****p* < 0.01, ***p* < 0.05, **p* < 0.1. All continuous variables are in logs

**Table 11:** Impact of mergers on branch density: Big mergers (> 500 branches)

Variables	(1)	(2)	(3)
Mergers (> 500 branches)	− 0.00223	− 0.00310	− 0.0146***
	(0.00233)	(0.00209)	(0.00455)
Constant	14.49***	− 9.733***	43.52***
	(0.617)	(0.908)	(3.840)
Observations	263,365	109,225	154,140
*R*^2^	0.308	0.252	0.192
Number of tracts	16,687	14,638	16,248
Tract FE	Yes	Yes	Yes
Year FE	Yes	Yes	Yes
County × Year FE	Yes	Yes	Yes
Sample	Treated tracts	Treated tracts	Treated tracts
Period	All	Pre-crisis	Post-crisis

Robust standard errors in parentheses and clustered at the tract level

****p* < 0.01, ***p* < 0.05, **p* < 0.1. All continuous variables are in logs

**Table 12:** Differences in socioeconomic characteristics between tracts exposed and un-exposed to mergers

Variables	All sample		Matched sample	
	Exposed tracts	Unexposed tracts	Exposed tracts	Unexposed tracts
Income	10.792	10.842	10.750	10.775
Population density	7.477	7.250	7.486	7.577
% higher education	0.337	0.327	0.341	0.351
% older	0.301	0.256	0.304	0.322
% minority	0.288	0.219	0.221	0.215

Income and population density are in logarithms

## Footnotes

1 Several recent studies have analyzed the relevance of bank size (Berger et al. 2005), distance to branch offices (Nguyen 2019), and the presence of community banks (Kowalik 2014; Berger et al. 2014; Jagtiani et al. 2016; and Jagtiani and Maingi 2019) in relation to access to credit by small business. Financial exclusion can also affect social capital and economic growth at the local level (Bruhn and Love 2014).

2 The US banking industry has undergone a number of waves of mergers of varying intensity. According to Elfakhani et al. (2003), “Banking analysts agree that increasing competition, deregulation, technological advancements, bank failures, cost savings, and the desire to form one-stop financial centers are among the major factors driving the continuing consolidation trend.”.

3 Allen et al. (2008), in an analysis of the impact of market structure on the diffusion of e-banking in Canada for the period 1998–2006, show that banks had more incentives to reduce the number of branches and to promote e-banking in regions where competition was more intense.

4 Studies on the initial adoption of online banking in the USA have shown that the Internet channel served as a complement to (rather than a substitute for) physical branches (DeYoung et al. 2007).

5 Data for this variable are available only for the period 2010–2017. The mean value of this variable in this period stands at 5.4%.

6 The effects of a bank’s characteristics on small business lending have been studied in other papers. Some show that stronger bank–borrower relationships are associated with better treatment for borrowers, in terms of lower interest rates and reduced collateral requirements (Berger and Udell 1995), increased credit availability (Petersen and Rajan 1995; Cole 1998) and more protection against interest rate shocks (Berlin and Mester 1998). Other papers have examined how lending practices differ between large and small banks, reporting that small banks tend to rely more on soft information (Cole 2004; Berger et al. 2005).

7 More generally, the relevance of soft information for lending to small firms is discussed in Petersen and Rajan (1995, 2002), Berger and Udell (1995), Cole (1998) and Beck and Demirguc-Kunt (2006).

8 In 1988, the Douglas amendment to the *Bank Holding Company Act* of 1956 prevented holding companies from acquiring out-of-state banks unless the state explicitly permitted such acquisitions. In practice, no state authorized acquisitions of this kind.

9 Copeland et al. (1995) record two waves of mergers in this period, in the mid-1960s and in the late 1980s extending until 1998. Elfakhani et al. (2003) explain that since then such waves have been frequent.

10 Rice and Strahan (2010) explain that between 1994 and 1997, the *Riegle-Neal Act* allowed states to regulate interstate branching in line with four provisions: (1) states were allowed to set minimum age requirements to determine when a bank could be acquired in an interstate merger; (2) bank entry via de novo interstate branching was only permitted if states allowed it explicitly; (3) states had to decide to legalize the acquisition of a single branch or a number of branches from other banks; and (4) states could impose deposit caps of less than 30%, which prevented interstate mergers for some banks.

11 This measure reversed the *Glass-Steagall Act* of 1933 and allowed commercial and investment banking activities to be conducted within the same financial company (De Bergia 2014).

12 Adams and Gramlich (2014) show that the number of new bank charters in the USA has declined in recent years. From 1990 to 2008, over 2000 new banks were founded, more than 100 per year. From 2009 to 2013, only seven new banks were created, fewer than 2 per year. Tranfaglia (2018), in a study of bank branch closures within the Philadelphia, Chicago and Baltimore metropolitan statistical areas (MSAs) in the period 2010–2016, reports evidence of clustering at short distances (2–5 km) in both Chicago and Philadelphia MSAs, but not in Baltimore. Banks maintain their branches in urban areas and reduce their presence in quasi-urban and rural areas.

13 The information is for 2020. Census tracts generally contain between 1,000 and 8,000 people with an optimum size of 4000. Their boundaries are delineated with the objective of their remaining stable over many decades, although the US census modifies their boundaries regularly. Also notice that our dataset excludes eight overseas states: American Samoa, the Federated States of Micronesia, Guam, the Marshall Islands, the Northern Mariana Islands, Palau, Puerto Rico, and the Virgin Islands.

14 We exclude branches that do not hold any deposits and entities that are not for commercial use or which provide a limited customer service. These offices represent around 4% of the FDIC dataset.

15 Note, information for 2012 is an interpolation of the years 2011 and 2013.

16 Fuster et al. (2019) define FinTech lenders as entities offering a mortgage application process that can be completed entirely online. These entities are stand-alone mortgage originators that primarily securitize mortgages and operate without deposit financing or a branch network.

17 Maudos and Vives (2019) analyze the banking system of the European Union during this period. They explain that the 2007–2009 crisis ended an expansionary period of the banking system and initiated a restructuring process that led to many mergers and acquisitions. Between 1997 and 2017, the number of credit institutions fell by 43% and the number of bank branches by 17%.

18 Several studies have shown that one of the justifications for mergers is cost reductions (DeYoung et al. 2009; Shen et al. 2020), yet they might also be motivated by such factors as monopoly power, raider valuation, empire-building and disturbance theory (Trautwein 1990).

19 Shen et al. (2020) explain that, during an economic crisis, mergers can generate three types of synergies for acquirers: (1) they obtain both revenue enhancements and cost reduction activities; (2) they acquire weak target banks at low prices, thus generating positive returns; and (3) they receive favorable terms, as regulators encourage mergers to prevent further bank failures and costly bailouts. In this sense, Evrensel (2008) examines the relationship between bank concentration and the hazard of bank failure in developed and developing countries. Moreover, Abreu et al. (2019) find evidence for European countries that state interventions in large banks after the financial crisis were consistent with the belief that these were “too big to fail”.

20 Our results are essentially unchanged when we consider a threshold of 100 branches.

21 The use of lags may only partly solve the endogeneity issue for the control variables. Endogeneity might alter the interpretation of the coefficients of these variables, but it should not affect the relationship between mergers and branch density, which is the main focus of our analysis. The use of instrumental variables would add noise to the analysis and omitting the variables would be worrisome.

22 Our analysis also reveals that in rural tracts there is a nonlinear relationship between branch density and population density: first branch density increases for low levels of population density and then it decreases. In urban tracts, branch density decreases with population density. See Figs. 6 and 7 in the Appendix.

23 The Census Bureau defines “urban areas” as either: (1) Urbanized areas, which contain 50,000 or more people; or (2) Urban clusters, which have at least 2500 people but fewer than 50,000 residents. Both urbanized areas and urban clusters are delineated primarily on the basis of population density. The Census Bureau also uses ‘land use’ divided by ‘land cover’ and distance criteria in assessing whether to include territory in an urban area. https://www.census.gov/content/dam/Census/library/publications/2020/acs/acs_rural_handbook_2020_ch01.pdf

24 Most studies define community banks as those that have a maximum asset size of $1 billion (FDIC 2012). Gilbert and Wheelock (2013) show that between 2001 and 2012, the share of rural county deposits held by community banks has changed little.

## References

[CR1] Abreu JF, Guerra Alves M, Gulamhussen MA (2019). State interventions to rescue banks during the global financial crisis. Int Rev Econ Financ.

[CR2] Adams RM, Gramlich JP (2014) Where are all the new banks? The role of regulatory burden in new charter creation. Federal Reserve Board Finance and Economics Discussion Series No. 2014–113

[CR3] Adams R, Amel D (2007) The effects of past entry, market consolidation, and expansion by incumbents on the probability of entry. Federal Reserve Board Working Paper Series

[CR4] Agarwal S, Hauswald R (2010). Distance and private information in lending. Rev Financ Stud.

[CR5] Aguirregabiria V, Clark R, Wang H (2016) The geographic flow of bank funding: branch networks and local-market competition. Mimeo

[CR6] Allen J, Clark R, Houde JF (2008) Market structure and the diffusion of e-commerce: Evidence from the retail banking industry. Bank of Canada Working Paper No. 2008–32

[CR7] Allen F, Carletti E, Cull R, Qian J, Senbet L, Valenzuela P (2014) Improving access to banking—evidence from Kenya. CEPR Discussion Paper No. DP9840. Center for Economic Policy Research, London

[CR8] Allen F, Demirgüç-Kunt A, Klapper L, Martinez Peria MS (2012) The foundations of financial inclusion: Understanding ownership and use of formal accounts. World Bank Policy Research Working Paper No. 6290

[CR9] Allen J, Evren Damar H, Martínez-Miera D (2016). Consumer bankruptcy, bank mergers and information. Rev Finance.

[CR10] Amel DF, Nellie Liang J (1997). Determinants of entry and profits in local banking markets. Rev Ind Organ.

[CR11] Aubuchon C, Wheelock DC (2010). The geographic distribution and characteristics of U.S. Bank Failures, 2007–2010: Do bank failures still reflect local economic conditions?. Federal Reserve Bank of St Louis Rev.

[CR12] Beck T, Martínez Peria MS (2010). Foreign bank acquisitions and outreach: evidence from Mexico. J Financ Intermed.

[CR13] Beck T, Demirguc-Kunt A (2006). Small and medium-size enterprises: access to finance as a growth constraint. J Bank Finance.

[CR15] Beck T, Ongena S, Şendeniz-Yüncüc I (2019). Keep walking? Geographical proximity, religion, and relationship banking. J Corp Finance.

[CR16] Berger AN, Cerqueiro G, Fabiana Penas M (2014). Market size structure and small business lending: Are crisis times different from normal times?. Rev Finance.

[CR17] Berger AN, Miller NH, Petersen MA, Rajan RG, Stein JC (2005). Does function follow organizational form? Evidence from the lending practices of large and small banks. J Financ Econ.

[CR18] Berger AN, Udell GF (1995). Relationship lending and lines of credit in small firm finance. J Bus.

[CR20] Berlin M, Mester L (1998). On the profitability and cost of relationship lending. J Bank Finance.

[CR21] Brown M, Guin B, Kirschenmann K (2016). Microfinance Banks and financial inclusion. Rev Finance.

[CR22] Bruhn M, Love I (2014). The real impact of improved access to finance: evidence from Mexico. J Finance.

[CR23] Calomiris C (2000). US bank deregulation in historical perspective.

[CR24] Carbo S, Gardener EPM, Molyneux P (2007). Financial exclusion in Europe. Public Money Manag.

[CR25] Celerier C, Matray A (2020) Bank-branch supply, financial inclusion and wealth accumulation. Rev Financ Stud, forthcoming

[CR27] Cohen AM, Mazzeo MJ (2010). Investment strategies and market structure: an empirical analysis of bank branching decisions. J Financ Serv Res.

[CR28] Cole RA (1998). The importance of relationships to the availability of credit. J Bank Finance.

[CR29] Cole RA (2004). Cookie-cutter vs. character: the microstructure of small business lending by large and small banks. J Financ Quant Anal.

[CR30] Copeland T, Koller T, Murrin J (1995). Valuation: measuring and managing the value of companies.

[CR555] De Bergia F (2014) La ley Glass-Steagall: origen, aplicación y derogación, Facultad de Ciencias Económicas y Empresariales Comillas ICAI/ICADE

[CR32] Degryse H, Ongena S (2005). Distance, lending relationships, and competition. J Finance.

[CR34] DeYoung R, Evanoff D, Moyneaux P (2009). Mergers and acquisitions of financial institutions: a review of the post-2000 literature. J Financ Serv Res.

[CR35] DeYoung R, Lang W, Nolle D (2007). How the Internet affects output and performance at community Banks. J Bank Finance.

[CR36] Elfakhani S, Ghantous RF, Baalbaki I (2003). Mega-mergers in the US banking industry. Appl Financ Econ.

[CR37] Ergungor OE (2010). Bank branch presence and access to credit in low- to moderate-income neighborhoods. J Money Credit Bank.

[CR38] Evrensel AY (2008). Banking crisis and financial structure: a survival-time analysis. Int Rev Econ Financ.

[CR39] FDIC (2012) DFIC community banking study. Federal Deposit Insurance Corporation

[CR41] FDIC (2018) 2017 FDIC National Survey of Unbanked and Underbanked Households. Federal Deposit Insurance Corporation. Division of Depositor and Consumer Protection ECONOMICINCLUSION.GOV

[CR42] FDIC QUARTERLY (2017) Factor Shapring recent trends in banking office structure for community and noncommunity banks. Federal Deposit Insurance Corporation

[CR43] Feng G, Serilitis A (2009). Efficiency, technical change, and returns to scale in large U.S. banks: panel data evidence from an output distance function satisfying theoretical regularity. J Bank Finance.

[CR44] Focarelli D, Panetta F (2003). Are mergers beneficial to consumers? Evidence from the market for bank deposits. Am Econ Rev.

[CR45] Fuster A, Plosser M, Schnabl P, Vickery J (2019). The role of technology in mortgage lending. Rev Financ Stud.

[CR46] Gilbert RA, Wheelock DC (2013). Big banks in small places: Are community banks being driven out of rural markets?. Federal Reserve Bank of St Louis Rev.

[CR47] Gropp R, Noth F, Schüwer U (2019) What drives banks' geographic expansion? The role of locally non-diversifiable risk. Mimeo

[CR49] Jagtiani J, Kotliar I, Maingi R (2016). Community banking mergers and their impact on small business lending. J Financ Stab.

[CR50] Jagtiani J, Maingi RQ (2019) How important are local community banks to small business lending? Evidence from mergers and acquisitions. Federal Reserve Bank of Philadelphia, Working Paper, WP 18–18

[CR51] Jayaratne J, Strahan PE (1996). The finance-growth nexus: evidence from bank branch deregulation. Quart J Econ.

[CR111] Johnson C, Rice T (2008) Assessing a decade of interstate bank branching. Washington Lee Law Rev 65(1):73–127

[CR52] Karceski J, Ongena S, Smith D (2005). The impact of bank consolidation on commercial borrower welfare. J Finance.

[CR54] Kowalik M (2014) Can small banks survive competition from large banks? Federal Reserve Bank of Boston, Work in progress

[CR56] Martin-Oliver A (2019). Financial exclusion and branch closures in Spain after the Great Recession. Reg Stud.

[CR57] McAllister PH, McManus D (1993). Resolving the scale efficiency puzzle in banking. J Bank Finance.

[CR58] McCord R, Prescott ES, Sablik T (2015) Explaining the decline in the number of banks since the Great Recession. Economic Brief, Federal Reserve Bank OF Richmond, EB15–03

[CR444] McLaughin S (1995) The impact of interstate banking and branching reform: evidence from the states. Current Issues in Economics and Finance 1, Federal Reserve Bank of New York

[CR59] Maudos J, Vives X (2019). Competition Policy in banking in the European Union. Rev Ind Organ.

[CR60] Nguyen HLQ (2014). Do bank branches still matter? The effect of closings on local economic outcomes.

[CR61] Nguyen HLQ (2019). Are credit markets still local? Evidence from bank branch closings. Am Econ J Appl Econ.

[CR62] Panetta F, Schivardi F, Shum M (2009). Do mergers improve information? Evidence from the loan market. J Money Credit Bank.

[CR63] Petersen MA, Rajan RG (1995). The effect of credit market competition on lending relationships. Quart J Econ.

[CR64] Petersen MA, Rajan RG (2002). Does distance still matter? The Information Revolution in Small Business Lending. J Finance.

[CR123] Rice T, Strahan P (2010) Does credit competition affect small-firm finance? J Finan 65(3):861–889

[CR65] Sapienza P (2002). The effects of banking mergers on loan contracts. J Financ.

[CR66] Sinclair SP (2001). Financial exclusion: an introductory survey.

[CR67] Shen C-H, Wu M-W, Chen T-H, Fang H (2016). To engage or not to engage in corporate social responsibility: empirical evidence from global banking sector. Econ Model.

[CR68] Shen C-H, Chen Y, Hsu H-H, Lin C-Y (2020). Banking crises and market timing: evidence from M&As in the banking sector. J Financ Serv Res.

[CR71] Tranfaglia A (2018) Shrinking networks: a spatial analysis of bank branch closure. Federal Reserve Bank of Philadelphia. WP 18–12

[CR72] Trautwein F (1990). Merger motives and merger prescriptions. Strateg Manag J.

[CR73] Vives X (2016). Competition and stability in banking: the role of regulation and competition policy.

[CR74] Vives X (2019). Competition and stability in modern banking: a post-crisis perspective. Int J Ind Organ.

[CR76] Wheelock DC, Wilson PW (2001). New evidence on returns to scale and product mix among U.S. commercial banks. J Monet Econ.

[CR77] Wheelock DC, Wilson PW (2001). Do large banks have lower costs? New estimates of returns to scale for U.S. Banks. J Money Credit Bank.

[CR78] Wheelock DC, Wilson PW (2004). Consolidation in US banking: Which banks engage in mergers?. Rev Financ Econ.

